# The impact of lake and reservoir parameterization on global streamflow simulation

**DOI:** 10.1016/j.jhydrol.2017.03.022

**Published:** 2017-05

**Authors:** Zuzanna Zajac, Beatriz Revilla-Romero, Peter Salamon, Peter Burek, Feyera A. Hirpa, Hylke Beck

**Affiliations:** a European Commission, Joint Research Centre, Directorate E – Space, Security and Migration, Via E. Fermi 2749, I-21027 Ispra (VA), Italy; b JBA Consulting, Skipton, Broughton Hall, Skipton BD23 3AE, UK; c International Institute of Applied Systems Analysis, Laxenburg A-2361, Austria; d University of Oxford, School of Geography and the Environment, Oxford, UK; e Princeton University, Civil and Environmental Engineering, Princeton, NJ, United States

**Keywords:** Global streamflow, Lakes, Reservoirs, LISFLOOD, Global sensitivity and uncertainty analyses, Parameterization

## Abstract

Lakes and reservoirs affect the timing and magnitude of streamflow, and are therefore essential hydrological model components, especially in the context of global flood forecasting. However, the parameterization of lake and reservoir routines on a global scale is subject to considerable uncertainty due to lack of information on lake hydrographic characteristics and reservoir operating rules. In this study we estimated the effect of lakes and reservoirs on global daily streamflow simulations of a spatially-distributed LISFLOOD hydrological model. We applied state-of-the-art global sensitivity and uncertainty analyses for selected catchments to examine the effect of uncertain lake and reservoir parameterization on model performance. Streamflow observations from 390 catchments around the globe and multiple performance measures were used to assess model performance.

Results indicate a considerable geographical variability in the lake and reservoir effects on the streamflow simulation. Nash-Sutcliffe Efficiency (NSE) and Kling-Gupta Efficiency (KGE) metrics improved for 65% and 38% of catchments respectively, with median skill score values of 0.16 and 0.2 while scores deteriorated for 28% and 52% of the catchments, with median values −0.09 and −0.16, respectively. The effect of reservoirs on extreme high flows was substantial and widespread in the global domain, while the effect of lakes was spatially limited to a few catchments. As indicated by global sensitivity analysis, parameter uncertainty substantially affected uncertainty of model performance. Reservoir parameters often contributed to this uncertainty, although the effect varied widely among catchments. The effect of reservoir parameters on model performance diminished with distance downstream of reservoirs in favor of other parameters, notably groundwater-related parameters and channel Manning’s roughness coefficient. This study underscores the importance of accounting for lakes and, especially, reservoirs and using appropriate parameterization in large-scale hydrological simulations.

## Highlights

• The effects of lakes and reservoirs on global daily streamflow are evaluated.
• Reservoirs affect model performance substantially in the global domain.
• Lakes’ effects on model performance are limited to few catchments.
• Lakes and reservoirs reduce return levels discharge thresholds globally.
• Reservoir parameters contribute to uncertainty of model performance metrics.

## 1 Introduction

Lakes and man-made reservoirs are key components of terrestrial hydrological systems. They affect flow regimes by changing the magnitude and timing of streamflow, usually by attenuating and delaying flows, but also through releases from reservoirs which can result in severe downstream floods. The impact of reservoirs on global streamflow has become considerable over the 20th century (Vörösmarty et al., 1997, Chao et al., 2008, Lettenmaier and Milly, 2009), during which the storage capacity of global reservoirs increased from less than 100 km^3^ in 1900 to approximately 8300 km^3^ in 2000 (Chao et al., 2008, ICOLD, 1998, 2007, 2009, 2011). The majority of large river systems around the world are fragmented by dams (Gao et al., 2012, Nilsson et al., 2005). The spatio-temporal quantification of the impacts of lakes and reservoirs is essential in terms of assessment of water-related hazards such as droughts and floods and hydrologic models may serve as essential tools for this purpose (Zhou et al., 2016, Oki and Kanae, 2006).

Some of the currently used global and continental scale hydrological models (GHMs; Bierkens, 2015, Bierkens et al., 2015, Döll et al., 2003, Coe, 2000, Meigh et al., 1999) that explicitly represent lakes and reservoirs, were used to assess the impacts of lakes and/or reservoirs on global- or regional-scale streamflow simulations (Biemans et al., 2011, Coe, 2000, Coe and Foley, 2001, Döll et al., 2009, Haddeland et al., 2006, Hanasaki et al., 2006, Meigh et al., 1999, Vörösmarty et al., 1997, Zhou et al., 2016). Above all, these previous studies highlighted the considerable impact of dams and reservoirs on the large-scale hydrological simulations. However, these studies mainly assessed the effect of dams on long-term (monthly – seasonal) streamflow, aggregated to catchment or regional scales. In this study we focus on estimating lake and reservoir effects on fully spatially distributed (at 0.1° resolution), daily streamflow simulations suited for global flood forecasting. Our overall objective is to improve streamflow simulations within the Global Flood Awareness System (GloFAS; Alfieri et al., 2013)—a probabilistic, medium-range flood forecasts at the global scale with a forecast horizon of 30 days (see www.globalfloods.eu). Within the GloFAS, the LISFLOOD hydrological model (De Roo et al., 2000, Van Der Knijff et al., 2010, Burek et al., 2013a) is used to simulate river routing and groundwater processes. The LISFLOOD lake and reservoir routines were developed specifically to provide realistic streamflow simulations at lakes and reservoirs outlets with a (sub-) daily time steps with the objective of improving flood forecasting for river sections downstream of large water bodies. These routines are parameterized with information contained within global-scale datasets, using a methodologically consistent approach, in order to avoid data bias due to political and geophysical boundaries (Arheimer et al., 2012). Although existing global inventories such as the Global Lakes and Wetlands Database (GLWD; Lehner and Döll, 2004) and the Global Reservoir and Dam Database (GRanD; Lehner et al., 2011) provide extensive metadata, some information necessary for parameterization and validation of lake and reservoir routines is not available. This includes for example descriptions of hydrographic conditions for lakes (e.g., outlet characteristics) and historical operation records for reservoirs. Openly shared reservoir records for deriving case-specific operation rules (and related model parameters) are only available in some developed countries (CEDEX, 2016, Gao et al., 2012, Hanasaki et al., 2006). We attempt to overcome these data limitations by relating some parameters to global-extent auxiliary data. For example, we estimate the outflow characteristics of lakes based on the channel width at the lake outlet, and we derive reservoir parameters based on simulated ‘naturalized’ streamflow. However, such an approach is associated with considerable uncertainty around parameter values which may adversely affect model performance.

To examine how uncertainty of lake and reservoir parameters propagates through the model and, as a result, affects model performance we use global sensitivity and uncertainty analyses (GSA/UA; Saltelli et al., 2004). River flow in sections downstream of lakes and reservoirs is controlled by a combination of factors relating to the natural variation of river flow and the lake and reservoir processes. GSA provides means of exploring the magnitude and spatial extent of influence of lake and reservoirs processes on the model response. Understanding the relative importance of lake and reservoir parameters is essential to advance global streamflow simulation. Our work has two specific objectives: 1) to quantify the effect of lakes and reservoirs on the performance and the extreme value statistics of the global daily streamflow simulations, and 2) to quantify the relative contributions of lake and reservoir parameters to the uncertainty.

## 2 Materials and methods

### 2.1 Modeling framework

#### 2.1.1 Hydrological modeling

**Fig. 1:**
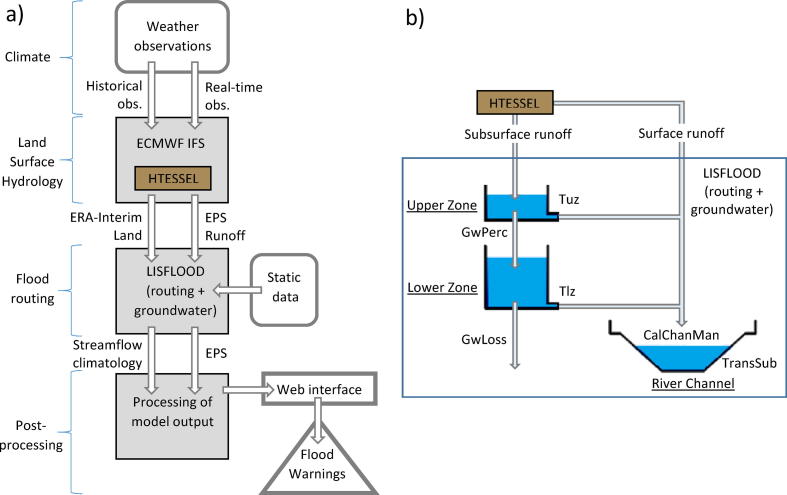
Schematic overview of the GloFAS modeling framework (from Alfieri et al., 2013, Revilla-Romero et al., 2015). (A) Overview of the GloFAS setup. The blue-contoured polygon indicates the input and output datasets and model; (B) Schematic of the LISFLOOD model). Light blue arrows in panel (B) represent water fluxes. The parameter names are explained in Table 1. (For interpretation of the references to colour in this figure legend, the reader is referred to the web version of this article.)

The hydrological simulations in GloFAS (Alfieri et al., 2013) were performed using a land surface scheme coupled to a river routing model (Fig. 1). The Hydrologically modified Tiled ECMWF Scheme for Surface Exchanges over Land (H-TESSEL; Balsamo et al., 2009) was used for generating surface and subsurface runoff, and a simplified version of the LISFLOOD hydrological model was used for flow routing and simulation of groundwater processes. LISFLOOD is a spatially distributed, partly conceptual and partly physically-based model, primarily developed to simulate major hydrological processes in large catchments (De Roo et al., 2000, Van Der Knijff et al., 2010). The simplified version of the model simulates groundwater processes and flow routing, human water use, and lakes and reservoirs. The daily global runoff fields produced by H-TESSEL were resampled from ∼80 km (see Section 2.2.3) to the LISFLOOD resolution of 0.1° (approximately 10 km at the equator), and routed using the kinematic wave approach (Chow et al., 1988) with a time sub-step of 4 h.

Spatial physiographic inputs were derived from various sources. Global river network and other river characteristics (e.g., flow direction, upstream area, and flow length) were taken from the global river network database of Wu et al. (2012), the river width map was taken from the Global Width Database for Large Rivers (GWD-LR; Yamazaki et al., 2014), while channel Manning’s roughness coefficient was calculated from land surface elevation and upstream area (De Roo et al., 2000, Burek et al., 2013a).

#### 2.1.2 Lake and reservoir routines

The lake routine simulates the outflow from lakes at each time step based on: (i) upstream inflow, (ii) precipitation over the lake, (iii) evaporation from the lake, (iv) the lake’s initial level, and (v) lakes outlet characteristics (defined by the *α* parameter which is derived based on the channel width at the lake outlet, following Burek et al. (2013a)). Groundwater flow (lateral or vertical) between lakes and surrounding aquifers is not simulated. The procedure is described in more detail in Appendix A.

Reservoir outflow is calculated based on: (i) upstream inflow, (ii) precipitation over the reservoir surface, (iii) evaporation from the reservoir, and (iv) reservoir-specific characteristics and operation rules, represented by a number of parameters. Specifically, the outflow is calculated following four different set of rules depending on the current filling fraction of a reservoir (described in Appendix A). The rules attempt to reach the desirable level, called the normal filling level, by promoting either recharge (if storage is below normal) or release (if storage is above normal). Moreover, the approach applied in the routine guarantees a minimum outflow (to sustain downstream riverine ecosystems) and a non-damaging outflow (to prevent overtopping of the dam). Parameterization of the reservoir routine requires the specification of: (i) the reservoir storage capacity, (ii) the three threshold filling levels (conservative storage limit, normal storage limit, and flood storage limit), and (iii) the three streamflow release thresholds (minimum, normal outflow, and non‐damaging outflow; Burek et al., 2013a). Values for the storage capacity were extracted from global datasets (see Section 2.2.1), while the threshold filling levels were estimated based on expert opinion and the streamflow release thresholds from naturalized simulations (see Appendix B).

### 2.2 Data

#### 2.2.1 Lakes and reservoirs dataset compilation

We used three datasets containing the characteristics and geographical distribution of global lakes and/or reservoirs: 1) the Global Lakes and Wetlands Database (GLWD; Lehner and Döll, 2004), which contains the largest lakes (area > 50 km^2^) and reservoirs (storage capacity ≥ 0.5 km^3^); 2) the Global Reservoir and Dam Database (GRanD; Lehner et al., 2011), which contains reservoirs with a storage capacity >0.1 km^3^, as well as many smaller ones; and 3) the World Register of Dams (WRD), compiled by the International Commission on Large Dams (ICOLD), which contains approximately 33,000 large dams (>15 m high) and associated metadata (ICOLD 1998, 2009).

**Fig. 2:**
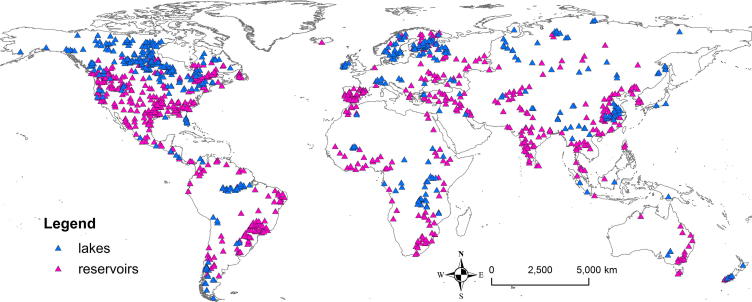
Locations of lakes and reservoirs within the global LISFLOOD model setup.

We incorporated in total 463 of the largest lakes and 667 largest reservoirs selected from GLWD and GRanD into the global model setup (Fig. 2), and we georeferenced them to the GloFAS river network. Since ICOLD does not provide geographical coordinates of dams (Lehner et al., 2011), the dataset was less useful for our purpose. As GLWD provides only shoreline polygons of lakes and reservoirs, the location of outlets on the river network was determined based on the shoreline polygons and the upstream area map. The lakes were required to have a surface area >100 km^2^ and had to be located on main river channels. Thus, we excluded many lakes that were either endorheic (e.g., Lake Chad in Nigeria), located on tributaries and seasonally fed by rivers (e.g., Lake Faguibine in the Niger catchment), or in the vicinity of the coast (e.g., Lake Izabal in Guatemala).

In the case of reservoirs, we included all of the world’s largest reservoirs from the GLWD, and in addition some reservoirs from the GranD, resulting in a total cumulative storage capacity of 4601 km^3^ which comprises approximately 65% of the total global large reservoir storage capacity (ICOLD, 2007, 2011).

**Fig. 3:**
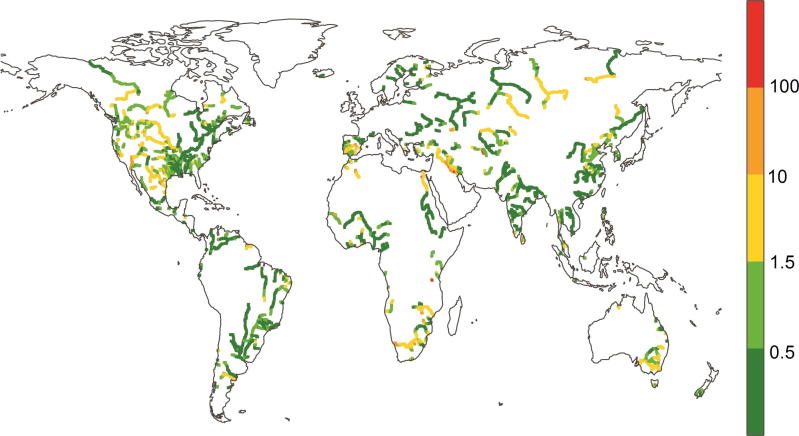
Spatially distributed c [–] ratio (reservoir volume to mean annual streamflow).

Fig. 3 illustrates the potential impact of reservoirs included in GloFAS on streamflow at global scale. To provide a continuous estimate of the potential reservoir effect along river reaches, the ratio (c [–]) of reservoir volume to mean annual discharge proposed by Nilsson et al. (2005) and Vörösmarty et al. (1997) was calculated for each grid cell. This ratio makes use of the upstream cumulative reservoir capacity [m^3^] and the cell-specific total volume of annual natural streamflow [m^3^]. Very high reservoir capacity to streamflow ratios can be found, for example, for the Euphrates in the Middle East (c > 100), the Oranje in Africa, and the Colorado in North America (c > 10), while medium impacted river sections (c > 1.5) are found for the Murray in Australia and most northern North American catchments. Conversely, very low reservoir capacity to streamflow ratios can be found, for example, for the Amazon and the Paraná in South America.

#### 2.2.2 Observed streamflow data

**Fig. 4:**
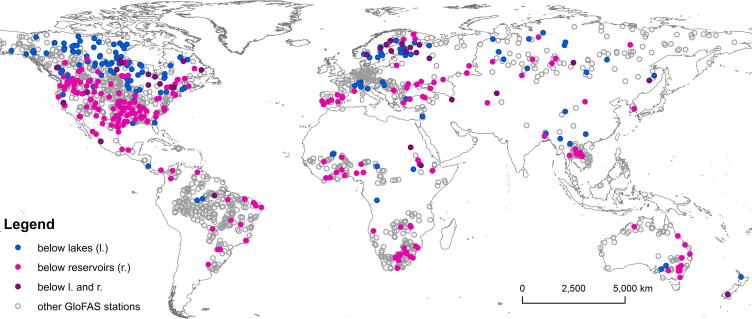
Location of GloFAS stations with daily streamflow observations.

Daily streamflow data were compiled from various sources, primarily the Global Runoff Data Centre (GRDC), complemented with information obtained from national providers for areas where the GRDC has few or no stations. We use daily observations for 390 stations (Fig. 4), located downstream of GloFAS lakes and/or reservoirs, with upstream area >10,000 km^2^ and at least 4 years of uninterrupted data during the simulation period (1980–2013).

#### 2.2.3 Meteorological input data

We used the ERA-Interim/Land dataset (Balsamo et al., 2015), which is based on the ERA-Interim reanalysis dataset (Dee et al., 2011), to force the hydrological model. The dataset consists of daily ∼80-km resolution fields of surface and subsurface runoff for global land areas for 1980–2014. The ERA-Interim precipitation was bias-corrected to match the monthly averages from Global Precipitation Climatology Project (GPCP; Huffman et al., 2009), a precipitation product based on merging satellite and gauge observations (Balsamo et al., 2015). Potential evaporation over open water bodies was estimated based on the surface radiation budget (Burek et al., 2013b) from ERA-Interim meteorological variables.

## 3 Evaluation methods

### 3.1 Assessing the effect of lakes and reservoirs

We assessed the model performance using historical streamflow data for the three alternative model configurations: (i) baseline scenario – neither lakes nor reservoirs included, (ii) lake scenario – only lakes included, and (iii) lake and reservoir scenario – both lakes and reservoirs included. For practical reasons the lakes and reservoirs were implemented into GloFAS using a stepwise procedure: first only lakes, secondly reservoirs added to lakes. Therefore, in this analysis the separate effect of reservoirs was not evaluated. For each configuration we ran the model for the 34-year period from 1980 to 2013 using the same initialization procedure and inputs. The first year was used as warm-up period and therefore excluded from evaluation of model performance.

#### 3.1.1 Effect of lakes and reservoirs on streamflow

We evaluated the model performance using daily streamflow observations. The performance metrics were calculated for the period with non-missing observed data during the simulation period. We considered multiple performance measures, including the Nash-Sutcliffe Efficiency (NSE; Nash and Sutcliffe, 1970), Kling-Gupta Efficiency (KGE; Gupta et al., 2009), Pearson linear correlation coefficient (r), and percent bias (PBIAS; Moriasi, 2007).

The normalized effect of the lake scenario as well as the lake and reservoir scenario for each performance measure was quantified as a skill score. For example, for the lake and reservoir scenario the skill (S) for KGE was defined as:

(1) $${\text{S}}_{\text{KGE}}=\frac{{\text{KGE}}_{\text{lakes and reservoirs}}-{\text{KGE}}_{\text{baseline}}}{{\text{KGE}}_{\text{optimal}}-{\text{KGE}}_{\text{baseline}}}$$

where: KGE_lakes and reservoirs_ represents the KGE for the scenario with lakes and reservoirs, KGE_baseline_ represents the KGE for the baseline scenario, and KGE_optimal_ represents the optimal value for KGE (KGE = 1). A positive skill score means that the performance has improved, whereas a negative skill score means that the performance has deteriorated.

#### 3.1.2 Effect of lakes and reservoirs on return levels

We quantified the effect of lakes and reservoirs on extreme flows (5- and 20-year return period levels). GloFAS defines the severity of a flood event relative to hydrological thresholds. Hence, determining accurate flood thresholds is important for skillful threshold exceedance forecasting (Alfieri et al., 2013, Hirpa et al., 2016). The Gumbel extreme-value distribution was fit to the daily annual maxima of streamflow to derive flood return levels for all three scenarios based on the simulated streamflow climatology for the 34-years period. The 5- and 20-year flood levels (Q5, and Q20, respectively) were estimated for each grid cell.

To compare the effect of lakes and reservoirs on the threshold values, we compared return levels for the lake and for the lake and reservoir scenarios to the baseline scenario as follows:

(2) $$\Delta \text{QL}=\left(\frac{{\text{QL}}_{\text{scenario}}-{\text{QL}}_{\text{baseline}}}{{\text{QL}}_{\text{baseline}}}\right)\text{,}$$

where ${\text{QL}}_{\text{scenario}}$ and ${\text{QL}}_{\text{baseline}}$ are return levels derived from the streamflow climatology of either lakes or lakes and reservoirs scenario and the baseline scenario, respectively, and L denotes the return period.

We compared the simulated return levels, with and without lakes and reservoirs, with point data obtained from extreme value analysis on observed discharge. As none of the stations included in the GloFAS database had continuous records of observed discharge for the entire simulation period (1980–2010), we used the reduced time window of 1996–2010. We evaluated normalized differences between simulated and observed return levels as:

(3) $$\Delta {\mathrm{QL}}^{\prime }=\left(\frac{{\mathrm{QL}}_{\mathrm{sim}}-{\mathrm{QL}}_{\mathrm{obs}}}{{\mathrm{QL}}_{\mathrm{obs}}}\right)$$

where QL_sim_ and QL_obs_ are return levels derived from the simulated streamflow climatology and observations, respectively, and L denotes the return period.

### 3.2 Global sensitivity and uncertainty analyses

**Fig. 5:**
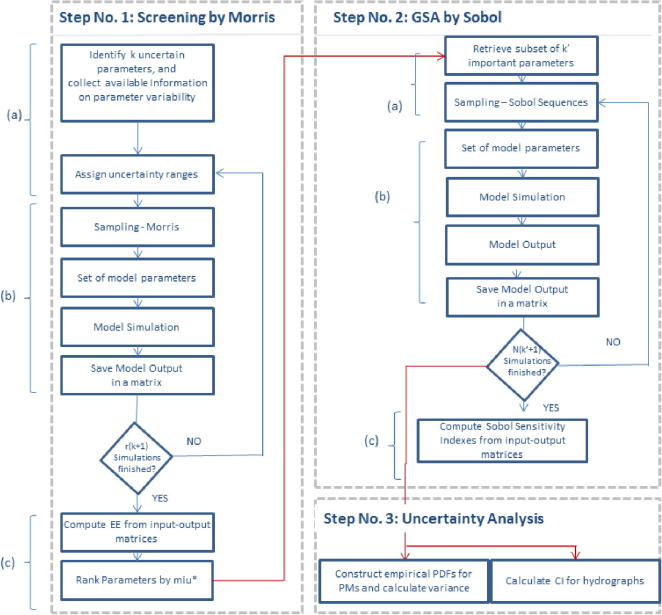
Schematic of the screening, Global Sensitivity Analysis (GSA), and Uncertainty Analysis (UA) procedures.

Global Sensitivity and Uncertainty Analyses (GSA/UA) were performed based on quasi-Monte Carlo (MC) simulations following the general outline proposed by Saltelli et al. (2012) as described in detail below. First, screening analysis (Saltelli and Annoni, 2010, Saltelli et al., 2012) was performed using the modified method of Morris (Campolongo et al., 2007; step 1 in Fig. 5). This step required a relatively small number of model evaluations to filter out non-important parameters. The parameters (also referred to as factors) identified as important were subsequently used for the next steps. Second, the variance-based GSA method of Sobol (Sobol, 1993, Sobol, 2001) was applied (step 2 in Fig. 5), and the quantitative sensitivity measures were calculated. Finally, uncertainty analysis was performed (step 3 in Fig. 5) using the same set of model simulations as used for step 2.

Description of the model parameters and corresponding ranges used for the GSA/UA is presented in Table 1. The parameter ranges were selected based on expert opinion as well as previous uncertainty assessments and calibration studies (e.g., FEYEN et al., 2008, Feyen et al., 2007).

**Table 1:** Model parameters used for GSA/UA including the uncertainty ranges.

	**Parameter**	**Process**	**Format**	**Default Value**	**Min**	**Max**	**Units**	**Description**
1	LakeMultiplier	Lakes	Scalar	1	0.3	3	–	Multiplier applied to calculate parameter alfa (lake outlets characteristics) from alfa = x * RW
2	rclim	Reservoirs	Table	0.1	0.05	0.15	–	Conservative storage fraction
3	rnlim		Table	0.3	0.3	0.7	–	Normal storage fraction
4	rflim		Table	0.97	0.8	0.99	–	Flood storage fraction
5	rminq		Table	0.05	0.05**	0.15**	m^3^/s	Minimum outflow
6	rnormq		Table	0.3	0.3**	0.7**	m^3^/s	Normal outflow
7	rndq		Table	0.97	0.9**	0.99**	m^3^/s	Non-damaging outflow

8	CCM	Channel routing	Scalar	1.5	0.1	15	–	Multiplier applied to Channel Manning’s n
9	CCM2		Scalar	3	0.1	15	–	Multiplier applied to Channel Manning's n for second routing line

10	UZTC	Groundwater	Scalar	10	1	40	[d mm^Gwα^]*	UpperZoneTimeConstant-time constant for water in upper zone
11	LZTC		Scalar	200	10	5000	[d]	LowerZoneTimeConstant-time constant for water in lower zone
12	GwPV		Scalar	0.5	0	2	[mm d^−1^]	Maximum rate of percolation going from the Upper to the Lower zone
13	GwL		Scalar	0	0	0.5	–	Maximum loss rate out of Lower response box, expressed as a fraction of lower zone outflow

* Gwα – parameter that defines the nonlinearity of the relation between the storage in and the outflow from the upper groundwater zone to the channel.

** Flows are expressed as percentiles of naturalized (without lakes and reservoirs) flow, these percentile values are subsequently converted into m^3^/s.

Within the method of Morris, the parameter sampling space is subdivided in a number of regularly spaced intervals at which local derivatives are obtained for a number r of sampling trajectories (Morris, 1991). Subsequently, two measures are calculated for each parameter, based on *r* local derivatives (i.e. elementary effects). A mean of absolute values of elementary effects (µ^∗^) estimates the overall importance of a given parameter, and a standard deviation of elementary effects (σ) estimates effects due to parameter interactions. The method provides a qualitative ranking of the parameters’ importance with respect to model output based on performance measures. For more details on the SA method, see Appendix B. The method of Sobol decomposes the total variance of the model output and quantifies the parameter contributions to the model output uncertainty (Saltelli et al., 2004, Saltelli et al., 2008). The parameter input space is sampled with Sobol sequences (Sobol, 2001). Two sensitivity measures were calculated for each parameter, using approximate MC integrations (Saltelli et al., 2008): 1) the first-order sensitivity index *S_i_* that measures the direct contribution of parameter *i* to the total output variance (Eq. (B1) in Appendix B), and the total sensitivity index *S_Ti_* that contains the sum of all effects involving parameter *i* (Eq. (B2)). Therefore the interaction effects can be isolated by calculating a remainder *S_Ti_* − *S_i_*. The empirical distributions for performance measures obtained from the MC simulations serve as a baseline for deriving UA measures, such as: variance, standard deviation (SD) or confidence intervals (CIs).

The above framework was implemented using R (R Core Team, 2011). The number of required runs for each step depends on a number of parameters (see Appendix B). A number of 140 simulations was required for the screening (for 13 parameters presented in Table 1), and approximately 10,000 simulations were performed for Sobol (for top 10 parameters identified by screening). Performing LISFLOOD computations for the global domain consumes considerable running time, therefore we limited the GSA/UA to 11 selected catchments, and used a simulation period of 4 years for the Morris analysis and 2 years for the Sobol analysis. The test catchments represent diverse hydro-meteorological conditions, and include: Niger and Nile in Africa; Amu Darya, Syr Darya, Pyasina and Lam Chi in Asia; Murray in Australia; Ebro and Guadiana in Europe; Colorado in North America, and Tocantins in South America.

## 4 Results and discussion

### 4.1 Assessing the effect of lakes and reservoirs

#### 4.1.1 Effect of lakes and reservoirs on streamflow performance

**Fig. 6:**
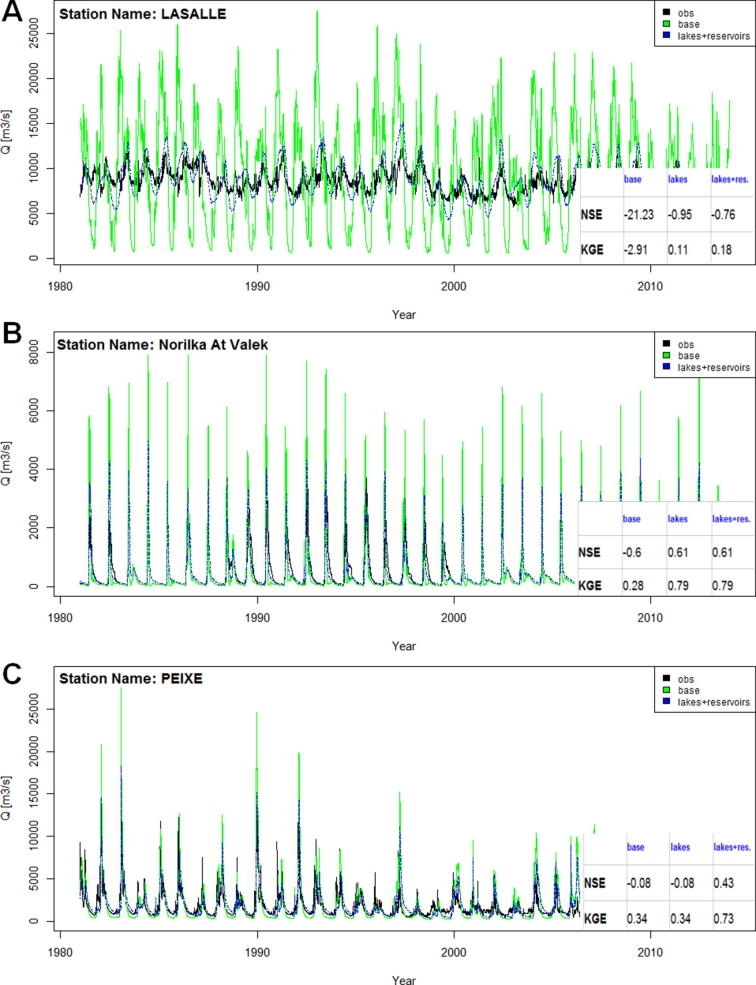
Example hydrographs and corresponding statistics showing effects of simulated lakes and reservoirs, for: (A) St. Lawrence (USA), (B) Pyasina (Russia), (C) Tocantins (Brazil).

The general observed effect of lakes on simulated streamflow was attenuation of peaks and delayed timing due to detention and evaporation. The relative importance of these processes for determining lake outflows varied for each individual catchment and was conditioned on lake surface area, climate, magnitude of upstream river inflows, as well as lakes’ outlet characteristics. We illustrate how the incorporation of lakes affects simulated streamflow for the Lasalle station, located on the St. Lawrence, downstream of Lake Ontario in the Great Lakes region (Fig. 6A). In this region the hydrology is strongly affected by open water evaporation (Spence et al., 2013). Therefore due to incorporation of lakes (and lake surface evaporation) the amplitude and magnitude of simulated streamflow improved at downstream river reaches. Similar effects were observed for other catchments with large-surface lakes, especially when located in areas prone to high potential evaporation (e.g., Tanganyika, Lake Victoria in Africa). On the other hand, small-surface lakes may affect streamflow mainly through detention. As observed for the station Valek at Norilka (Russia, Fig. 6B) upstream lakes considerably improved timing and magnitude of peak flows. In addition, reservoirs exerted a considerable influence on simulated hydrographs through anthropogenic operating rules that altered natural distribution of streamflow. We illustrate this effect in Fig. 6C for the Peixe station on the Tocantins. The incorporation of the Serra da Mesa reservoir, which is mainly used for hydropower, markedly affected the seasonal distribution of water from the wet summer to the dry winter, and improved the simulated hydrographs for downstream stations.

**Fig. 7:**
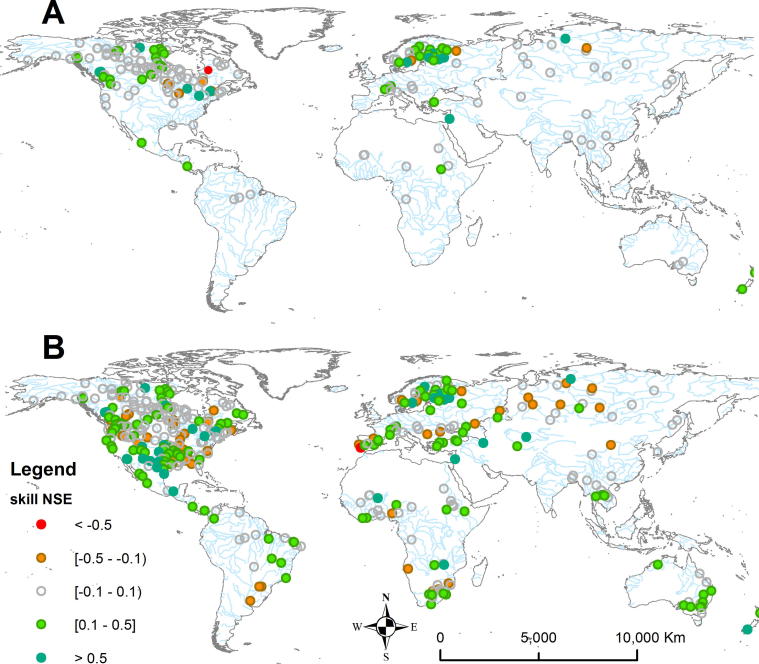
Normalized change of skill (NSE) for GloFAS stations after incorporation of: (A) lakes (for 171 stations located downstream of lakes), (B) lakes and reservoirs (for 390 stations located downstream of lakes or reservoirs).

The inclusion of lakes generally improved the streamflow simulation skill. However, for most catchments the effect of lakes on skill scores was rather small, with a few notable exceptions. Fig. 7A shows the spatial distribution of NSE skill scores obtained for simulations with the lake scenario as compared to the baseline scenario. For 171 catchments downstream of lakes, the introduction of lakes improved the NSE for 67% of the catchments with a median improvement of 0.09 (mean 0.21, maximum 0.96), while the NSE worsened for 22% of the catchments with a median deterioration of −0.04 (mean −0.07, maximum −0.34). The scores remained the same for 11% of stations downstream of lakes. The KGE skill improved for 41% catchments with a median improvement of 0.2, while it worsened for 37% catchments with a median deterioration of −0.05. The most pronounced improvements were seen for several catchments in Europe (notably the Rhine, Rhone, Po), and Yukon and Fraser rivers. The limited effect of lakes, even for some lake-abundant catchments (e.g., Amazon and Ob), may be explained by the fact that for these basins many lakes are positioned on the tributaries, therefore the influence of lakes on a daily variability of streamflow in main channels (where the gauge stations are generally located) is minor.

The lake and reservoir scenario resulted in changes in NSE skill scores for numerous catchments (Fig. 7B), indicating that model performance is more strongly affected by reservoirs than lakes. For the 390 catchments with lakes or reservoirs (including 253 catchments affected only by reservoirs) the NSE improved for 65% of the catchments, with a median improvement of 0.16 (mean 0.24, maximum 0.96). Model performance deteriorated for 28% of catchments with a median deterioration of −0.09 (mean −0.11, maximum −0.95). The KGE performance improved for 38% catchments with a median increase of 0.2, while it deteriorated for 52% of catchments with a median deterioration of −0.16. Substantial improvements with the introduction of reservoirs were found for many catchments, including Zambezi (Africa), Mekong, Amu Darya, and Syr Darya (Asia), Murray (Australia), Tocantins and Magdalena (South America), Columbia, Colorado, and Rio Bravo (North America), among others. However, the effect of including reservoirs was not always beneficial as scores deteriorated for some stations, many of them located in North America (e.g., the Apalachicola and Alabama, and sections of the Mississippi), but also for other catchments, such as Oranje (Africa), Ob (Asia), Danube (Europe), as well as, Rio Iguaçu (South America). In most cases, the deterioration was rather small, such as for the Danube, where the introduction of only one reservoir—Iron Gates 1—resulted in a slightly lower NSE for all downstream stations.

**Fig. 8:**
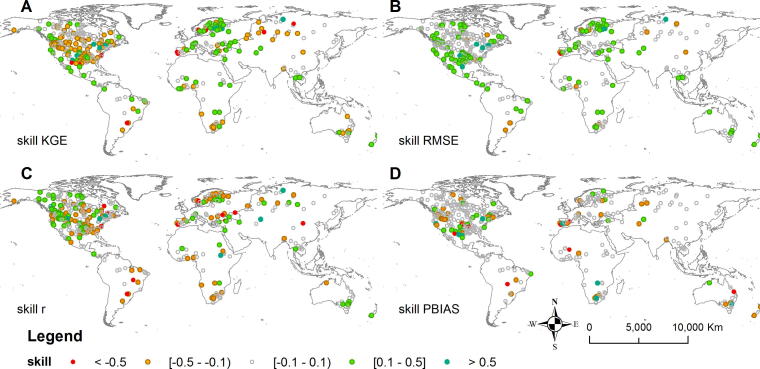
Normalized change of skills for GloFAS stations after incorporation of lakes and reservoirs: (A) KGE, (B) RMSE, (C) r, (D) PBIAS. Only stations located downstream of lakes or reservoirs are presented.

The maps for the other performance measures (Fig. 8) show deterioration of r and PBIAS values mainly in North America. The accurate representation of timing and bias components is challenging for this region, which is heavily impacted by anthropogenic alterations not represented by the model (e.g., inter- and intra-catchment water transfers and joint reservoir operations). Furthermore, the reservoir routine, in its current form, does not account for downstream water demands or water abstractions from reservoirs. It is rather assumed that reservoirs are operated mainly for flood control. In reality reservoirs may also be operated for other purposes (e.g., irrigation, hydropower) or multiple purposes (e.g., flood control during the wet season, irrigation during the dry season). Reservoirs operated for irrigation exhibit seasonal release patterns, depending on downstream crop water demands. The ongoing developments of the LISFLOOD include incorporation of such demands into the reservoir routine. The deterioration of skill scores may also result from the unrepresentativeness of the default reservoir parameter values as a result of lack of knowledge regarding the specific reservoir operating rules. The sensitivity to the parameter values is further investigated in the Section 4.2.1. Furthermore, decisions such as preventive water releases before anticipated heavy rainfalls (e.g., before typhoons) are difficult to represent in global hydrological models. Due to the unavailability of streamflow data for many regions (e.g., India, vast areas of east China, and South America) we were unable to evaluate the effect of lakes and reservoirs on model performance for these regions.

#### 4.1.2 Effect of lakes and reservoirs on return levels

**Fig. 9:**
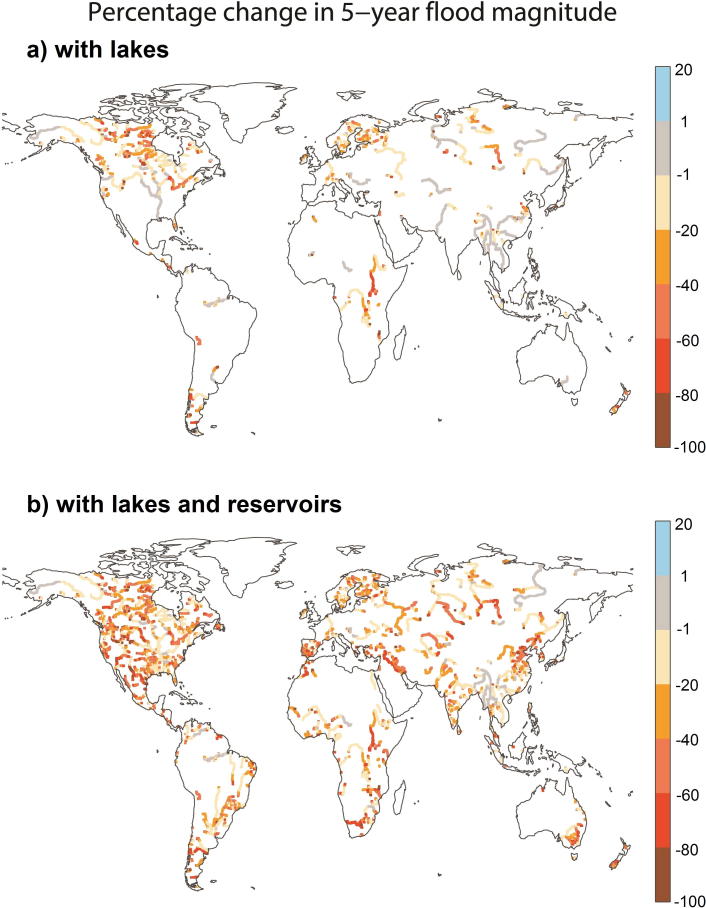
The normalized differences for 5-year return levels as a result of introduction of lakes (A) and both lakes and reservoirs (B) into the global simulations (see Eq. (2)). The locations of incorporated lakes and reservoirs are shown in Fig. 2.

Fig. 9 shows for the entire land surface the reduction in 5-year return level (Q5) with the introduction of only lakes (Fig. 9A) and both lakes and reservoirs (Fig. 9B) compared to the baseline scenario. At a global scale, the effect of lakes on Q5 is spatially limited to few catchments. The catchments with streamflow regimes strongly affected by lakes include Yenisei, Pyasina, and Amur in Asia, Rhine and Po in Europe, Saint Lawrence in North America, and rivers in Patagonia (e.g., Santa Cruz) in South America. In Africa, the effect of lakes on Q5 was most prominent for the Nile, showing a 75% decrease downstream Lake Victoria, for the upper Congo (e.g., the Lukuga river downstream of Lake Tanganyika) and the Shire.

Reservoirs have a pronounced effect on Q5 in many catchments worldwide (Fig. 9B). In North America Q5 was reduced by as much as 80% for some rivers (e.g., the Rio Bravo and Colorado Rivers) with changes observed for all major rivers. In South America, reservoir effects were visible for some major rivers, such as the Parana, Tocantins, Sao Francisco, and Negro. Also in Australia, in contrast to lakes which had a negligible effect, reservoirs had a substantial impact on Q5. In the Murray catchment, for example, Q5 was reduced by >50% for many river sections. In some African catchments (for example in the Niger, Zambezi, Orange, and Senegal) Q5 was substantially impacted by reservoirs. For the Nile, the effect of the Aswan dam was clearly visible for the section below Lake Nasser. Furthermore, reservoirs effects are evident for some catchments in the Middle East. In the Euphrates catchment Q5 was reduced by approximately 80% for most river sections, while in the Tigris catchment Q5 was reduced by >60% for selected sections. Asian catchments with considerably altered Q5 due to reservoirs include Amu Darya and especially Syr Darya (where Q5 was reduced by up to 60%), as well as the Ganga, Godavari, Krishna (India), Indus (Tibet, India, Pakistan), Irtysh, Yenisei (Russia), Yongding He, Yellow River, Huai He, and Liao He (China). Similar impacts were observed for Q20.

**Fig. 10:**
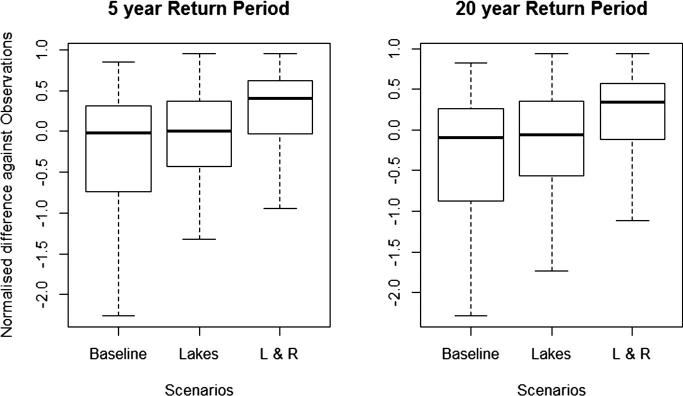
Normalized difference between discharge levels obtained from simulations and observations for 5-year and 20-year return periods.

The comparison of simulated return levels with and without lakes and reservoirs, with point data obtained from extreme value analysis on observed discharge indicated that incorporating lakes generally improved the representation of extremes, as summarized in Fig. 10. The buffering effect of lakes resulted in a narrower range of normalized differences of Q5 and Q20 between simulations and observations. The inclusion of reservoirs resulted in further reductions in the range, but also in more frequent overestimation of simulated return levels, as compared to observations. This shift could indicate that our current parameterization of reservoirs for critical conditions does not reflect real-world operating rules (i.e., values of critical storage and critical discharge might be too low) resulting in insufficient reduction of simulated peak flows for downstream stations.

In summary, the results show that lakes and especially reservoirs exert an important influence on streamflow dynamics in many catchments. This is in agreement with several previous studies (Biemans et al., 2011, Coe, 2000, Döll et al., 2009, Haddeland et al., 2006, Hanasaki et al., 2006, Meigh et al., 1999, Vörösmarty et al., 1997, Zhou et al., 2016), although we assessed daily simulations at global scale and also investigated the impact of lakes separately. Our results highlight the importance of accounting for lakes and reservoirs in hydrological model applications, particularly when focusing on streamflow extremes as for agricultural planning, or flood and drought forecasting and monitoring.

### 4.2 Global sensitivity and uncertainty analyses

#### 4.2.1 Uncertainty analyses

In this study, we assessed the impact of uncertainty in lake and reservoir parameters (among other parameters) on hydrological model performance. Other sources of uncertainty include model structure initial and boundary conditions, forcing data (e.g., meteorological inputs), and static input data (Beven, 2006, Engeland et al., 2016, Pappenberger et al., 2005, Sperna Weiland et al., 2015), but these are not considered here. While, it is generally agreed that rainfall forecasts (Fekete et al., 2004, Nasonova et al., 2011, Pappenberger et al., 2005, Pappenberger et al., 2011, Sperna Weiland et al., 2015) and initial conditions (Yossef et al., 2013) represent the main source of uncertainty of hydrological simulations, parameter uncertainty may also exert important effect on model performance and predicting extreme events globally (Chaney et al., 2015).

**Fig. 11:**
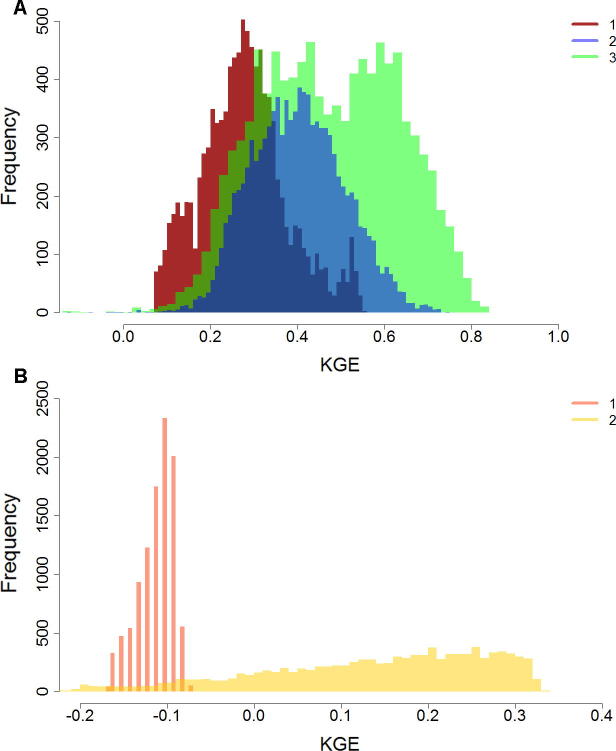
Empirical distributions for KGE for selected stations for: (A) Tocantins; (B) Amu Darya. Numbers in the legend correspond to station identifiers, used in Table 2 and Fig. 12.

**Fig. 12:**
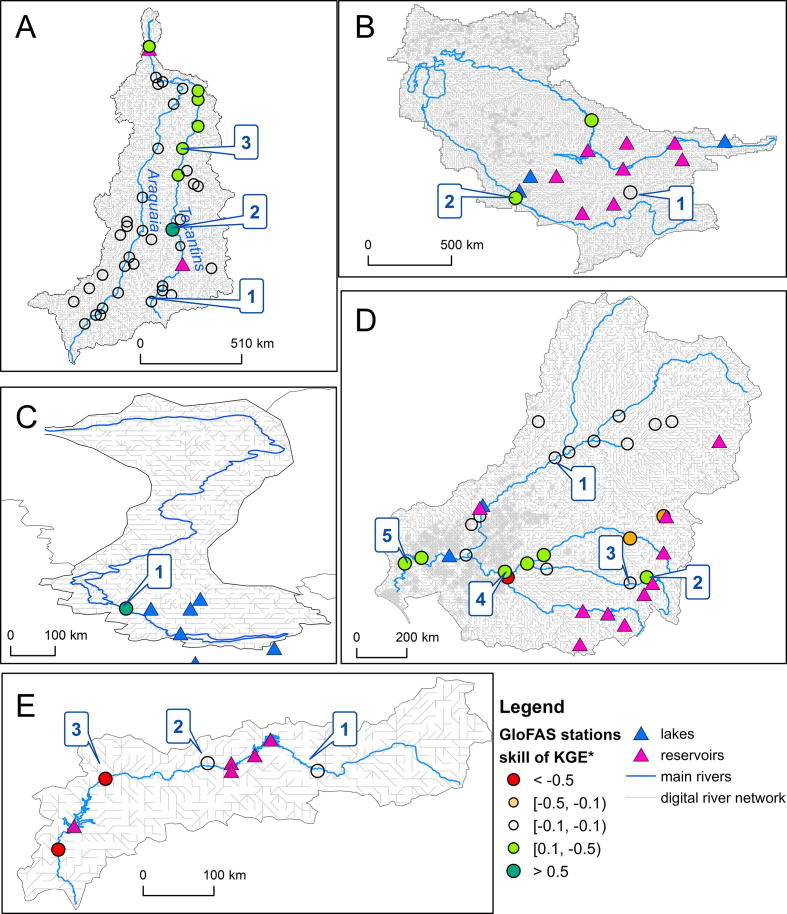
Test catchments with locations of GloFAS stations: (A) Tocantins, (B) Amu Darya, (C) Pyasina, (D) Murray, (E) Guadiana. Colors of circles indicate change of KGE skill after incorporation of lakes and reservoirs. (For interpretation of the references to colour in this figure legend, the reader is referred to the web version of this article.)

The UA results for test catchments revealed a considerable uncertainty of model performance propagated from parameters. Fig. 11A illustrates UA results for the example stations in the Tocantins basin (locations presented in Fig. 12A). Empirical distribution of KGE, obtained from MC simulations, are characterized with standard deviation (SD) that ranges from 0.10 to 0.16 for locations 1 and 3, respectively, with corresponding 95% confidence intervals (obtained from 2.5 and 97.5 percentile values) of [0.10, 0.52] and [0.20, 0.76], respectively. Thus, alternative parameter sets, used as a baseline for MC simulations, may lead to very different values of model performance metrics, and subsequent decisions regarding model performance. Uncertainty measures were spatially variable within test catchments, with small uncertainty observed only for a few stations with consistently poor model performance (see for example, Amu Darya in Fig. 11B) possibly due to underestimated runoff, provided by the rainfall runoff model. Considerable uncertainty of KGE scores, observed for most of the test catchments, illustrates the potential of enhancing model performance by formal parameter estimation, with a focus on sensitive parameters. The contribution of lake and reservoir parameters to this uncertainty is presented in the next section.

#### 4.2.2 Sensitivity analyses

The routing parameter CCM (multiplier applied to channel Manning’s n) appears to exert strong influence overall on the streamflow performance measures, especially in the more arid catchments (e.g., Murray and Colorado). KGE, r, and NSE are highly sensitive to this parameter which affects the timing and magnitude of peak flows. For some of the test catchments (e.g., Niger, Nile, Lam Chi, and Tocantins) model performance is to a large degree controlled by groundwater processes, and the groundwater parameters (GwL, GwPV, UZTC, and LZTC, defined in Table 1) are the most important in terms of KGE response to the parameter variations (Table 2). The effect of CCM for these catchments is pronounced, with its importance tending to increase for downstream reaches. LISFLOOD uses kinematic wave approach for river routing. Although this is best feasible approach for flood routing at a global scale (as dynamic wave is too data demanding) it may involve some disadvantages. Typically flood peaks in lowland reaches could be simulated too fast, arriving one or few days earlier than in reality. This could explain a typically high importance of CCM in affecting performance metrics for downstream river reaches, as this parameter can greatly influence timing of the flood wave.

**Table 2:** Morris ranking for KGE according to µ^*^ for selected test catchments and stations. Only ranks for sensitive parameters are shown. The colors represent the different ranks. The locations of stations are shown in Fig. 12, letters A–E before catchment name correspond to catchment maps, numbers in square brackets correspond to selected locations.

For the majority of test catchments, the effect of reservoir parameters is strongest for the river sections downstream of reservoirs. This effect is reduced with increasing distance downstream, in favor of other parameters. Spatial patterns indicating local influences of reservoir parameters were observed for some test catchments (Amu Darya, Murray, Ebro, Guadiana, and Tocantins, Table 2 and Fig. 12). For example within Tocantins catchment reservoir parameters rnlim and rnormq were highly important (3rd and 4th in the ranking) for location 2, below the reservoir, while these effects were diminished, further downstream, in favor of groundwater parameters and CCM (Table 2, Fig. 12A). However, some other test catchments, such as Nile, Niger, or LamChi exhibited very limited or no sensitivity of the hydrological response to reservoir parameters. One possible explanation for such limited effect of reservoir parameters is that LISFLOOD model with the current setup tends to overestimate water balance for these catchments, and therefore factors that reduce amount of water in the system (i.e. groundwater parameters) are the most influential ones in affecting performance metrics. This example illustrates the potential of GSA to serve as a tool to examine model behavior and indicate problematic areas in the domain. Possibly the processes that reduce amount of water in the river systems (e.g. evaporation from channel, channel seepage) are currently represented suboptimally, especially for basins with extensive wetlands (e.g. Nile, Niger, Mekong). Prospective enhancements of transmission loss module of the LISFLOOD model, which include seasonal changes of the open water surface of river systems, are expected to improve estimation of water balance for wetland areas.

Among the reservoir factors, parameters related to normal operating conditions: normal storage fraction (rnlim) and normal outflow (rnormq) are generally the most important; rnlim may locally replace CCM and groundwater parameters in their importance ranks (Table 2). Other reservoir parameters are also of significance for some stations with exception of minimum outflow (rminq) that has generally negligible effect. The importance of rnlim and rnormq may derive from the fact that most often reservoir operations take place under normal conditions, and/or relatively wide uncertainty range associated with specification of these parameter. Contrary to reservoirs factors, the effect of lake parameter (LM) was negligible for catchments with lakes (e.g.: Nile, Niger and Murray). The only exception, among the test catchments, was observed for the Pyasina, where performance measures were mostly affected by LM, followed by CCM, and groundwater parameters (Table 2).

**Fig. 13:**
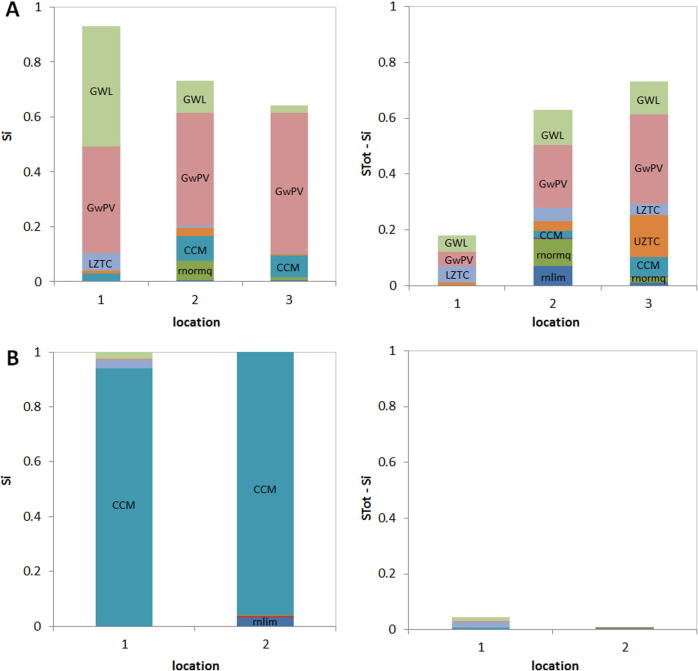
GSA results for selected locations for example catchments: (A) Tocantins, B) Amu Darya. The graphs on the left show first order effects (S_i_), while the graphs on the right show interaction effects (S_Tot_ − S_i_).

The screening analysis for the test catchments indicated that the number of parameters identified as important was effectively smaller than the full set of 13 parameters considered for GSA/UA (Table 2). In some cases it was reduced to two or even one parameter. Both SA methods (Morris and Sobol) were consistent in identifying a set of important parameters; however we identified two main differences that may occur between screening and Sobol results: Morris may identify a wider set of important parameters, and the shifts of parameter importance between two methods. As seen for example for station Peixe (Fig. 13A) the quantified contribution of reservoir parameters rrflim, rndq (identified as important by Morris) was negligible (below 1% cutoff value). On the other hand, a significant contribution (7% of the total KGE variance) of the reservoir parameter rnormq was confirmed. For another example station Kerki in Amu Darya rflim, indicated as important by Morris, contributed marginally (<1%) to the KGE variance (Fig. 13B), while the KGE uncertainty was dominated by CCM (97%) with a small contribution of rnlim. A narrower set of factors identified as important by Sobol may be explained by the fact that the Morris method is susceptible to type I - false positive - errors (that is identifying a not-important factor as important) (Saltelli et al., 2004). However, such behavior should not underline the value of screening in narrowing the set of important parameters, as the number of potentially important factors is much reduced during screening for most of the cases. The shifts of parameter importance between two methods, apart from methodological differences, could possibly be attributed to the fact that different simulation time periods were used for screening and GSA (4 versus 2 years). We acknowledge this potential limitation of the present study. As both methods provide similar identification of important parameters, and Sobol is associated with high computational requirements, Morris may be considered for application of the SA on the global-scale.

The SA results are useful for model evaluation and development as they identify priorities for parameters’ refinements, for example by means of additional data collection, calibration, or regionalization. The results of the analyses presented here could serve as a guideline for selecting catchment-specific parameter sets to calibrate the latest LISFLOOD global version, using both river gauges as well as satellite data (as tested in Revilla-Romero et al., 2015). With reduced parameter sets, effective (i.e. better estimates) and efficient (i.e. faster computational speed) optimization algorithm could be achieved. In particular, the reduced computational time is a critical priority for the global-scale model calibration. Further estimates of reservoir parameters could be improved by calibration of daily reservoir releases or storages, simulated by LISFOOD, against historical reservoir records. However, for this purpose an open access database of daily reservoir records (currently non-existing) would be indispensable. Possibly, over time a compiled database of observed streamflow, as used here, could be extended to store reservoir discharge and storage records from the available sources. Furthermore, the sensitivity and uncertainty analyses, as presented here, could serve as a starting point for a future assimilation of products of satellite radar altimeters into the global LISFLOOD. Incorporation of satellite derived lake and reservoir surface levels could compensate for the lack of ground reservoir records, and improve model skill, especially for areas with highest uncertainty, due to lakes and reservoirs parameters.

## 5 Conclusions

In this study, we estimated the effect of lake and reservoir and their parameterization on daily river streamflow simulations of a global spatially-distributed hydrological model. Streamflow observations from 390 catchments around the globe were used for evaluation of the LISFLOOD model performance. Incorporation of lakes resulted in improvement of model performance for few catchments globally, but where present, the improvement was substantial. On the other hand, incorporation of reservoirs improved performance for many regions. However, for some catchments, mainly in North America, the timing component of simulated hydrographs deteriorated. The challenges related to global scale reproduction of daily reservoir streamflow were mostly identified as variability of individual reservoirs operating rules, and limited reservoir records for reservoirs’ parameter estimation on the global scale.

The effect of lakes and reservoirs on simulating extreme flows was a reduction of the threshold levels for return periods of five and twenty years for the majority of the global domain. While inclusion of lakes generally improved representation of extreme discharge levels, inclusion of reservoirs resulted in a general tendency to overestimate extreme streamflow (as compared to base or lake scenarios).

Moreover, we applied global sensitivity and uncertainty analyses to examine the effect of lake and reservoir parameter uncertainty on model performance. The uncertainty analysis results revealed a considerable uncertainty of model performance metrics propagated from parameters. The sensitivity analysis identified the Manning’s channel multiplier (CCM), and groundwater parameters as the most sensitive factors in the currently-tested LISFLOOD model set-up. The CCM very often controlled model performance for drier basins, and downstream river sections, while groundwater factors were more important for wet catchments or for locations where the model overestimates discharge. The lake parameter was generally of limited importance, even for test catchments with several lakes; whereas the reservoir parameters (mainly related to normal operating conditions) had a pronounced effect for river section downstream of dams; this effect was reduced further downstream, in favor of other parameters.

Morris and Sobol identified a consistent subset of important parameters, indicating the usefulness of the method of Morris for computationally demanding models. SA using the method of Morris (due its low computational requirements) could be used to explore large scale hydrological model applications, such as LISFLOOD global used in this study, and to limit the dimensionality of parameters prior to model calibration. Ultimately, a global open access database of daily reservoir records would be indispensable to further enhance the simulation of any hydrological model when including reservoir dynamics.

## Appendix A Lakes and reservoir routines of LISFLOOD

The lake and reservoir routines of LISFLOOD model are described in Burek et al., 2013a, Burek et al., 2013b. Both lakes and reservoirs are simulated as points on the channel network.

### A.1 Lakes

The change in lake storage (*S*) over a time period of $\Delta t=t_{2}-t_{1}$ can be related to the average inflow ($Q_{\text{in}})$, average outflow ($Q_{\text{out}}$, total accumulated precipitation (*P*) and net evaporation (*E*) from the lake area (*A*) over the time step as Eq. (A1).

(A1) $$\frac{S_{2}-S_{1}}{\Delta t}=\frac{\left(Q_{\text{in}1}+Q_{\text{in}2}\right)}{2}-\frac{\left(Q_{\text{out}1}+Q_{\text{out}2}\right)}{2}+\frac{(P-E)\;*\;A}{\Delta t}$$

Most of the terms in Eq. (A1) are known from previous time step ($S_{1}\text{,}Q_{\text{out}1}\;\text{and}\;Q_{\text{in}1}$), meteorological data (e.g., *P* and *E*), lake properties (lake area), or from inflows from upstream area ($Q_{\text{in}2}$). However, two quantities need to be estimated: the lake storage ($S_{2})$ and outflow ($Q_{\text{out}2)}$ at the current time step. Rearranging Eq. (A1) to put the known quantities into one side gives Eq. (A2).

(A2) $$\frac{S_{2}}{\Delta t}+\frac{Q_{\text{out}2}}{2}=\frac{S_{1}}{\Delta t}+\frac{\left(Q_{\text{in}1}+Q_{\text{in}2}\right)}{2}-\frac{Q_{\text{out}1}}{2}+\frac{(P-E)\;*\;A}{\Delta t}$$

The lake outflow is related to the lake level (*H*) using the well-known weir equation of Poleni (Bollrich, 1992), assuming the weir is of parabolic shape:

(A3) $$Q_{\text{out}2}=\alpha \times H^{2}$$

where: [Q] = [m^3^s^−1^], *α* is a lake parameter defining lake outlet characteristics [ms^−1^], H is a lake level a.s.l. [m].

The lake effective storage (i.e. storage above sea level) can be related to the lake level using a simplified linear equation as:

(A4) $$S_{2}=A\times H$$

By combining Eqs. (A3), (A4), (A2), the lake outflow can be defined as:

(A5) $$Q_{\text{out}2}={\left(\sqrt{2\;*\;\mathrm{SI}+{\mathrm{LF}}^{2}}-\mathrm{LF}\right)}^{2}$$

where, $\mathrm{LF}=\frac{A}{\Delta t\sqrt{\alpha }}$, is a lake factor and *SI* is the right hand side of Eq. (A2).

The initial lake levels were estimated using average annual net inflow from the baseline simulations.

### A.2 Reservoirs

For reservoirs, the outflow is calculated with four different set of rules (Eqs. (A6), (A7), (A8), (A9)) depending on the current filling level of a reservoir.

(A6) $$Q_{\mathrm{res}}=\min\left(Q_{\min}\text{,}\frac{1}{\Delta t}F\times S\right)\;F⩽2\times L_{c}$$

(A7) $$Q_{\mathrm{res}}=Q_{\min}+(Q_{\mathrm{norm}}-Q_{\min})\frac{\left(F-2L_{c}\right)}{\left(L_{n}-2L_{c}\right)}\;L_{n}⩾F>2L_{c}$$

(A8) $$Q_{\mathrm{res}}=Q_{\mathrm{norm}}+\frac{\left(F-L_{n}\right)}{L_{f}-L_{n}}\times \max\left\{\left(I_{\mathrm{res}}+\frac{(P-E)\;*\;A}{\Delta t}-Q_{\mathrm{norm}}\right)\text{,}(Q_{\mathrm{nd}}-Q_{\mathrm{norm}})\right\}\;L_{f}⩾F>L_{n}$$

(A9) $$Q_{\mathrm{res}}=\text{max}\left(\frac{\left(F-L_{f}\right)}{\Delta t}\times S\text{,}Q_{\mathrm{nd}}\right)\;F>L_{f}$$

where the reservoir parameters are defined as follows; S: design storage capacity; $Q_{\mathrm{res}}$: outflow; F: fill fraction;$L_{c}$: conservative storage limit;$L_{n}$: normal storage limit; $L_{f}$: flood storage limit; $Q_{\min}$: minimum outflow; $Q_{\mathrm{norm}}$: normal outflow; $Q_{\mathrm{nd}}$: non-damaging outflow; $I_{\mathrm{res}}$: reservoir inflow; *P*: precipitation on the reservoir and *E*: evaporation from the reservoir.

As we lacked consistent, global operational records that could be used to derive reservoir parameters, we assumed the minimum, normal and maximum storage as 0.1, 0.3, and 0.97 fraction of total installed capacity, respectively. While the minimum, normal and non‐damaging outflow were assumed to correspond to the 5th, 30th, and 97th percentiles of naturalized daily streamflow (for the LTR), respectively. These parameter estimates are expected to have a various level of correctness for individual reservoirs.

## Appendix B Sensitivity analysis methods

### B.1 Screening with a method of Morris

The method is based on computing for each input a number of incremental ratios, called elementary effects (EEs), which are then averaged to assess the overall importance of a given input factor. Elementary effects are calculated by varying one parameter at a time across a discrete number of levels (p) in the space of input factors. The elementary effect is calculated from:

(B1) $${\mathrm{EE}}_{i}=\frac{f(x_{i}\text{,}\ldots \text{,}x_{i}+\Delta \text{,}\ldots \text{,}x_{k})-f(X)}{\Delta }$$

where: EE_i_ – elementary effect for a given factor Xi, Δ is a value in {1/(p − 1), 1–1/(p − 1)} this value defines a “jump” in the parameter distribution between two levels considered for calculating the elementary effect, p – number of levels.

Based on elementary effects calculated for each input factor, two sensitivity measures are proposed by Morris (1991): (1) the mean of distribution of absolute values of the elementary effects, µ^∗^, which estimates the overall effect of the parameter on a given output (Campolongo et al., 2007); and (2) the standard deviation of the effects, σ, which estimates the higher-order characteristics of the parameter (such as curvatures and interactions).

(B2) $$\mu_{i}^{*}=\frac{1}{r}\overset{r}{\underset{j=1}{\sum }}{\mathrm{EE}}_{i}^{j}$$

(B3) $$\sigma_{i}=\sqrt{\frac{1}{r}\overset{r}{\underset{j=1}{\sum }}{\left({\mathrm{EE}}_{i}^{j}-\mu_{i}\right)}^{2}}$$

where: r – number of elementary effects for parameter i (equivalent to a number of search trajectories), EE_i_ – elementary effect for parameter i.

Previous studies have demonstrated that using p = 4 and r = 10 produces satisfactory results (Campolongo et al., 1999, Saltelli et al., 2000), and we used these values in this study. The 10 best (mostly spread) search trajectories were selected from 500 pre-generated ones.

### B.2 Sobol – for variance-based SA

The variance-based method of Sobol (1993) that decomposes the total variance of model output between the different uncertain input factors and their interactions, according to the equation (Saltelli et al., 2004):

(B4) $$\text{V(Y)=}\underset{\text{i}}{\sum }{\text{V}}_{\text{i}}+\underset{\text{i < j}}{\sum }{\text{V}}_{\text{ij}}+\underset{\text{i < j < m}}{\sum }{\text{V}}_{\text{ijm}}+\ldots +V_{12...\text{k}}$$

where: V(Y) is the total variance of the model output Y, Vi is the fraction of the output variance explained by the ith model input factor, V_ij_ is the fraction of the variance due to interactions between factors i and j, and k is the number of inputs. The first order sensitivity index Si is calculated from the ratio of the output variance explained by the ith model input (V_i_) to the total output unconditional variance (V):

(B5) $${\text{S}}_{\text{i}}=\frac{{\text{V}}_{\text{i}}}{\text{V(Y)}}$$

Assuming the factors are independent, the total order sensitivity index S_Ti_ is calculated as the sum of the first order index and all higher order indices of a given parameter. For example, for parameter X_i_:

(B6) $${\text{S}}_{\text{Ti}}=1-\frac{{\text{V}}_{-\text{i}}}{\text{V}(\text{Y})}$$

where S_Ti_ is the total order sensitivity, and V_−i_ is the average variance that results from all parameters, except X_i_. Sobol indexes are constrained as follows: 0 ≤ S_i_ ≤ S_Ti_ ≤ 1.

While the method of Morris requires M = r(k + 1) model simulations, where: k – number of parameters, r – number of sampling trajectories (usually r = 10 is sufficient and provides satisfactory results). The method of Sobol (1993), as implemented in this study, requires M = (2k + 2)N model simulations (Lilburne and Tarantola, 2009), where k is the number of parameters, N is the number of samples (we used N = 512 as suggested by Lilburne and Tarantola (2009), and M is the total number of iterations.
